# Association between Hashimoto’s thyroiditis and papillary thyroid carcinoma: a retrospective analysis of 305 patients

**DOI:** 10.1186/s12902-019-0351-x

**Published:** 2019-05-29

**Authors:** Giuseppa Graceffa, Renato Patrone, Salvatore Vieni, Silvia Campanella, Sergio Calamia, Iole Laise, Giovanni Conzo, Mario Latteri, Calogero Cipolla

**Affiliations:** 1 Department of Surgical Oncological and Oral Sciences, University of Palermo, Via del Vespro, 129 90127 Palermo, Italy; 2 Division of General and Oncologic Surgery - Department of Cardiothoracic Sciences, University of Campania “Luigi Vanvitelli”, Via Pansini 5, 80131 Naples, Italy

**Keywords:** Hashimoto’s thyroiditis, Papillary thyroid carcinoma

## Abstract

**Background:**

The association between Hashimoto’s thyroiditis (HT) and papillary thyroid carcinoma (PTC) is a controversial question that is still under debate, its pathological significance and the eventual clinical implications of this association remaining unclear.

**Methods:**

The data regarding 305 patients were retrospectively analyzed. The patients were divided in two different groups. A first group made up of 142 patients undergoing surgery for differentiated thyroid carcinoma was compared to a control group of 142 analogous subjects operated for normofunctioning goiter. A second group was made up of 163 patients who had undergone total thyroidectomy (TT) with pre-operative diagnosis of HT.

**Results:**

In the first group of patients an association with HT was found in 28,6% of the patients with final histopathological diagnosis of PTC versus 7,7% of the patients with histopathological diagnosis of multinodular goiter, which was a significant difference (*p* <  0.001). In the second group, the association with PTC was found in 43 (40,2%) cases of HT nodular variant and in 3 cases (8,1%) of HT diffuse variant (*p* <  0.001).

**Conclusions:**

The relationship between HT and PTC is still far from clear and represents an unresolved issue. Our own study has underlined the frequent coexistence of these two pathologies, an aspect not to be neglected in clinical practice. Patients receiving HT diagnosis should undergo careful follow-up and, especially those with the nodular variant, should undergo a frequent both clinical and cytological evaluation of the nodular lesions, taking always into great consideration the surgical approach of total thyroidectomy.

## Background

Hashimoto’s thyroiditis (HT) is the most common autoimmune inflammatory pathology of the thyroid and is the main cause of autoimmune hypothyroidism. It is characterized by an infiltrate of immune cells able to determine the destruction of the gland, its fibrous involution and consequent hypothyroidism [1–3]. It was first described by Hakaru Hashimoto in 1912 as “lymphomatous struma” [4]. Global occurrence of HT is estimated between 0.3 and 1.5 cases out of 1000 individuals per year, prevalently among members of the female sex (5/20,1) between 30 and 50 years of age [5–7].

There are two different clinical variants: the diffuse form and the nodular form. The nodular form is dominated by a non-homogeneous thyroid parenchyma that presents fibrosis, sclerosis and calcifications and is the one primarily associated with neoplastic formations, papillary carcinoma of the thyroid in particular. Treatment is almost always medical, surgery is indicated in increase glandular volume with compressive symptoms, in unsatisfactory drug therapy and in suspicious of neoplastic degeneration of one or more nodules.

The link between HT and papillary carcinoma of the thyroid (PTC), first described by Dailey et al. in 1955 [5], is a controversial question. Despite the fact that this link was subject of numerous studies, there isn’t a unanimous scientific literature opinion and the medical debate is still open.

The primary outcome of this study was try to clarify if this link exists and how important it is. The secondary outcome was to identify its possible clinical, diagnostic and therapeutic implication.

## Methods

After approval by the institutional review board at University Hospital AUOP Paolo Giaccone of Palermo, a retrospective analysis of the medical records of a series of 305 consecutive patients was conducted. The patients were selected among 2175 undergone total thyroidectomy (TT) in the period from January 2006 to December 2016. All patients with preoperative diagnosis of both thyroid carcinoma and of Hashimoto’s thyroiditis were considered eligible for the study. Two hundred fifty-eight patients (84,6%) were females, 47 (15,4%) were male with 1/5 M/F ratio. The mean age was of 50,6 years (range 13–82). The 305 patients were divided in two different groups.

The first group (*Group 1*) consisted of 142 patients underwent TT for a TIR 5 preoperative fine-needle aspiration cytological diagnosis; the second group (*Group 2*) consisted of 163 patients underwent TT for a preoperative diagnosis of HT. The first group of patients was compared to a control group matched for sex and gender, consisting of 142 patients who had undergone surgical intervention during the same period involving total thyroidectomy for normal functioning multi-nodular goiter.

In all cases the preoperative diagnostic plan included thyroid hormones assay, thyroid ultrasonography, thyroid scintigraphy, and ultrasound-guided cytological examination using fine needle aspiration biopsy (FNAB). The diagnosis of HT was based on an increase in serum values of anti peroxidase and antithyroglobulin antibodies.

The Italian consensus to classify thyroid cytology has provided a standardized reporting scheme, including the subdivision of indeterminate for malignancy TIR-3 category into TIR-3A (low-risk) and TIR-3B (high-risk) [8].

The histopathological diagnosis of HT was based on the presence of diffuse, chronic, inflammatory infiltrate, mainly composed of T-lymphocytes and plasma cells organized in germinative centers, but also due to the presence of fibrotic areas, which did not extend beyond the capsule, as well as to the presence of atrophic follicles with numerous Hürthle cells and enlarged thyroid cells, characterized by abundant cytoplasm, which was eosinophil and rich in mitochondria.

ASA score ≤ III, age less than 82 years old, HT diagnosis, TIR5 preoperative cytological diagnosis and a complete clinical, laboratory and radiological evaluation were the main surgical inclusion criteria. All patients underwent total thyroidectomy.

Statistical analysis was performed by X^2^ test, with Yates’ correction where appropriate, a *P* value of less than 0.5 indicated statistical significance.

## Results

### Group 1

The 74,6% of all patients enrolled in this group were women (106 patients) with 1/3 M/F ratio; the mean age was of 50,2 years (range 13–82 years). PTC was confirmed at histological examination in 126 patients (88,7%); follicular carcinoma was diagnosed in 8 cases (5,6%), medullary carcinoma in 2 cases (1,4%), an oncocytic variant was found in 5 cases (3,5%) while 1 case (0,7%) was an insular variant.

This group of patients was compared to an analogous group matched for sex and gender composed of 142 patients who underwent surgical intervention during the same period involving TT for normal functioning multinodular goiter.

Of the142 patients who had undergone TT for thyroid carcinoma, in 36 cases thyroid histological examination showed the concomitant presence of a chronic lymphocytic thyroiditis to be attributed to HT. The link was found exclusively in the 126 cases with histological diagnosis of PTC with an incidence rate of association between PTC and HT equal to 28,6%. In 3 cases (2,3%), PTC was found in association with Graves’s disease and with hyper-functioning multi-nodular goiter. In the remaining cases, PTC either presented as a single nodule in a healthy gland or was associated with a histopathology of normal functioning multinodular goiter. Although, in the control group, the concomitant presence of a histopathology compatible with HT was found only in 11 cases (7,7%).

The association between PTC and HT has proved to be widely significant (*p* < 0.001) compared with that observed in patients who had undergone surgery for multinodular goiter (Table 1)*.*

**Table 1:** Papillary thyroid carcinoma coexistent with Hashimoto’s thyroiditis *(Group 1)*

	PTC (126 patients)	Multinodular goiter (142 patients)	
Mean age	51,3 yrs (range 27–71 yrs)	50,9 yrs (range 30–71 yrs)	NS
Sex:			
Male	30	32	NS
Female	96	110	
M/F ratio	1/3	1/3	
Euthyroid	126 (100%)	142 (100%)	NS
Coexistent HT	36/126 patients 28,6%	11/142 patients 7,7%	*P* < 0.001

### Group 2

The 93,2% of 163 patients were female (152 patients), 11 were male; the M/F ratio was of 1:14. The mean age was of 50,6 years (range 20–82 years). One hundred and seven cases (65,4%) were nodular variant of HT, 37 cases (22,7%) were diffuse variant of HT, in 19 cases (11,7%) HT had developed in patients during follow-up procedures for normal functioning multinodular goiter.

In HT nodular variant, 42 of 107 patients (39,2%) underwent surgical approach for a TIR 3B or TIR 4 preoperative cytological diagnosis, 53 cases (49,5%) because of a volumetric increase of one or more nodules, in 12 cases (11,2%) for the onset of local neck symptoms.

The 37 patients with diffuse HT variant and the 19 patients with HT resulting from previous goiter underwent surgical approach due to a volumetric increase of the gland, associated with a compressive symptoms.

Of the 163 patients who underwent TT with preoperative diagnosis of HT, in 51 cases (31,3%) at histopathological examination a differentiated thyroid carcinoma was found. In 47 cases (92,2%) it was PTC, in 3 cases (5,9%) follicular carcinoma and just in one case (1,9%) mucous-epidermoid carcinoma. A PTC was co-existent in 43 out of 107 cases (40,2%) of nodular variant HT, in 3 out 37 cases (8,1%) of diffuse variant HT and in 1 of 19 cases (5,2%) of HT which had developed on a previous goiter.

The average diameter of the tumours, established at histopathological examination, was of 10,6 mm (range: 0,2–20,6 mm) in 13 cases of PTC it was a question of microcarcinoma, including those 3 cases associated with diffuse variant HT, where the diameter of the PTC was ≤5 mm. In no case were found lymph nodes involvement or presence of distant metastasis. Among the remaining 112 patients with preoperative diagnosis of HT, in 13 cases (7,9%) an association with micro- and/or macrofollicular adenoma was present, 32 cases (19,6%) were associated with diffuse goiter and 44 cases (26,9%) with a normal functioning multinodular goiter. In the remaining 23 cases (14,1%) HT was not associated with any other pathology.

It is important to underline that 43 out of the 47 cases of PTC (91,5%) were found in patients with nodular variant HT compared with other forms of thyroiditis (*p* < 0.001), indicating a significantly higher risk of the PTC development on the nodular variant of HT.

The sex and age of patients nor the time lapse occurring between the diagnosis of HT and surgery seemed to be at significant at all. In fact, in the group of 47 patients with an association between HT and PTC, the male/female ratio was 1:12 and the mean age was 47,5 years, while in the 116 patients in whom HT was not associated with PTC, the male/female ratio was 2:22 and the mean age was 49,7 years. In patients who underwent surgery for HT associated with PTC, the HT diagnosis had been made on average 29,7 months before surgery (range: 0–96 months), whereas in patients with HT not associated with PTC, the diagnosis of HT had been made on average 31,3 months before surgery (range: 0–113 months) (Table 2).

**Table 2:** Hashimoto’s thyroiditis coexistent with Papillary thyroid carcinoma (Group 2)

	HT Nodular variant	HT Diffuse variant	
Coexistent PTC	43/107 patients 40,2%	3/37patients 8,1%	*P* < 0.001
	Coexistent HT and PTC	HT	
Mean age	47,5 yrs	49,7 yrs	NS
M/F ratio	1:12	2:22	NS
Time laps between HT diagnosis and surgery	29.7 m	31.3 m	NS

## Discussion

The association of HT with PTC has remained an active focus of research and controversy since it was first described in the 50’s. The aim of our study was to verify the association between HT and PTC by evaluating two groups of patients who underwent surgery at our institute between 2006 and 2016. In the first group, we compared the different histopathological pictures indicating HT found in patients undergoing surgery for thyroid carcinoma and in a control group involving patients who had undergone surgery for normal functioning goiter. In the second group of patients, we evaluated the incidence of PTC found in patients receiving surgery after a pre-operative diagnosis of HT. A significant association between HT and PTC was found in both groups of patients. In fact, in the first group of studied patients, a histological picture matching with HT was observed in 28,6% of cases undergoing surgery for PTC as opposed to the 7,7% of cases undergoing surgery for nodular goiter. In the second group of studied patients, in 31,3% of cases undergoing surgery for HT, the coexistence with PTC was revealed and in almost all cases it was a nodular variant of HT.

These findings, which confirm what reported in our own earlier study published in 2005 [9], are in agreement with various studies published in the literature [10–12] and, albeit indirectly, they suggest the presence of a link between the two pathologies, even though the true nature of such link is till today, topic of much discussion and debate.

The debate focuses particularly on the possible role of HT as a risk factor in the development of PTC or, alternatively, on its eventual role of defense against neoplasia and therefore as a factor for favorable prognosis.

Virchow had already hypothesized the presence of a pathogenetic link between inflammation and neoplastic degeneration in 1863. A study by Chui et al. [13] demonstrated that the follicular epithelium of the thyroid in the context of autoimmune thyreopathy is not homogeneous and that chronically present phlogistic infiltrates may in fact be responsible for the dysplastic transformation of the follicular epithelium. This would lead to the formation of so-called “zones of follicular displasia”, areas characterized by intense proliferation of follicular cells which present high concentrations of TG, TTF-1, HBM1, galectine3 and CK-19, even though the typical characteristics of papillary carcinoma are not present. This is a pre-neoplastic lesion, which can easily complete its evolution towards the form of PTC. This result confirms what was reported by other authors, who have found that the incidence rate of carcinoma in patients with HT range between 0.5 and 53% and it is much higher than that in patients without HT [14–16]. Moreover, in about 90% of HT cases the expression of the mutation of oncogenes RET/PTC, typical of PTC, was found in quantities that easily overlap between the two forms of pathologies [17–19].

At the same time, the expression of protein p63 appears to have a role in the correlation between HT and PTC. Unger et al. [20] had demonstrated that the expression of protein p63 appears to be greatly superior in patients with HT and PTC compared to those with a healthy thyroid, or with nodular goiter or follicular adenomas.

According to more recent studies, the coexistence of HT and PTC seems to find a further explanation in the conspicuous production of cytokines, which characterizes HT. These cytokines, whose number increases with phlogosis, would regulate the activation of pathways fundamental to neoplastic growth such as angiogenesis, cellular proliferation and the inhibition of apoptosis. In this respect, TNFα, the endothelial growth factor (VEGF), the nitric oxide produced by macrophages and the HMGB-1 protein seem to be the most involved cytokines [21–23].

Further new studies aim at underlying the importance of HT as a favorable factor in the prognosis of subjects with PTC. The coexistence of HT and PTC appears to be associated to clinical-pathological characteristics of reduced tumor aggressiveness and with diminished recurrence of the pathology [24–28]*.* Lun et al. [29] had produced a case-controlled study on 2478 patients who underwent TT. If compared with a group of patients with multi-nodular goiter, patients with PTC presented greater coexistence of HT; moreover, patients with HT associated PTC were younger, female, with smaller tumors and, in general, with a less advanced stage of TNM compared to those who had PTC without HT.

An effective prognosis for patients with PTC coexistent with HT is an important point when reflecting on the pathogenetic link between these two pathologies. It is well known that innate immune response constitutes the first mechanism of defense against agents, which would attack the organism, including tumor cells. Tumor cells are able to trigger both innate and non-innate immune response that could be responsible for the development of autoimmune phenomena leading to a completely new mechanism, that of autoimmunity due to an anti-neoplastic immune response.

The study conducted by Kari et al. [30] on mice models susceptible to thyroid disease supports this theory, showing how genetic predisposition to autoimmune thyroid disease may favor the appearance of an autoimmune response during an immune response to the tumor. This mechanism of autoimmunity associated to antitumor immune response appears to reveal itself in other studies too, such as in those aimed at analyzing the association between antitumoural immunotherapy and autoimmunity [31, 32].

The autoimmune response within the context of an antitumor immune response would be confirmed by the presence of anti-TG (AAT) and anti-TPO (ATPO) antibodies in about 20% of patients with thyroid carcinoma, with values twice higher in patients with PTC, compared to the general population [33]. The development of autoantibodies might be part of a more complex defense mechanism against tumors with the objective of eliminating the precursors of future neoplastic cells [34].

The reasons for an antitumor immune response might be found in the existence of papillary thyroid microcarcinoma which has not yet been diagnosed. This small neoplasia would be able to induce an antitumor immune response, followed by local phlogosis, which would promote the cross-reactivity and consequent epitope-specific autoimmune-response.

## Conclusions

In conclusion, the relationship between HT and PTC is still far from clear and represents an unanswered question particularly concerning the physiopathological and clinical mechanisms linking these two pathologies. Our own study has underlined the frequent coexistence of these two pathologies, and, unlike previous studies, it corroborates the most recent theories according to which the link between HT and PTC may be due to the presence of an autoimmune-response which develops along with an antitumoural immune-response. Nevertheless, further studies will be necessary, in order to comprehend the true nature of the link existing between the two pathologies.

In any case, regardless of how numerous the question marks may be, we cannot ignore the elevated rates of incidence of PTC in patients with HT. Accordingly, patients receiving HT diagnosis should undergo careful follow-up with periodical evaluation of thyroid hormones and autoantibodies and special attention paid to TSH and AAT. Especially those patients with the nodular variant should undergo both clinical and cytological evaluation of the nodular lesions. Moreover, given the greater risk of developing PTC (1.6 times greater), an adequate therapeutic planning should be in force for patients with nodular variant HT. The use of L4-Thyroxine has proved excellent in suppressing TSH and in reducing the risk of PTC. However, in certain conditions, especially in cases in which the indications noted above reach elevated concentrations, the surgical approach of TT must be given serious consideration.

## Abbreviations

AAT: Anti-thyroglobulin
ATPO: Anti-thyroperoxidase
FNAB: Fine needle aspiration biopsy
HT: Hashimoto’s thyroiditis
PTC: Papillary thyroid carcinoma
TRAb: Thyrotropin receptor antibodies
TT: Total thyroidectomy
